# New Binary Blends of Ethylene-co-n-butyl Acrylate (EBA) Copolymer and Low Molecular Weight Rosin Ester Resin with Potential as Pressure Sensitive Adhesives

**DOI:** 10.3390/ma11102037

**Published:** 2018-10-19

**Authors:** Sara Sancho-Querol, Andrés Jesús Yáñez-Pacios, José Miguel Martín-Martínez

**Affiliations:** Adhesion and Adhesives Laboratory, University of Alicante, 03080 Alicante, Spain; sara.sancho@ua.es or sarasanchoquero@gmail.com (S.S.-Q.); andresjesus.yanez@ua.es or andresjyp@gmail.com (A.J.Y.-P.)

**Keywords:** ethylene-co-n-butyl acrylate copolymer, glycerol rosin ester, pressure sensitive adhesive, compatibility, tack, adhesion

## Abstract

For improving the adhesion property of ethylene-co-n-butyl acrylate copolymer (EBA) at ambient temperature, binary blends of EBA with 27 wt% n-butyl acrylate and different amounts (20–62 wt%) of low molecular weight hydrogenated glycerol rosin ester (ECH) resin have been prepared. The addition of glycerol rosin ester resin decreased the crystallinity and size of the ethylene domains of the EBA copolymer. The addition of up to 50 wt% (100 phr) ECH resin improved the compatibility with the EBA copolymer, whereas when more than 50 wt% (100 phr) ECH resin was added, the compatibility of the blends did not change but the viscoelastic properties were noticeably decreased. Furthermore, the compatibility was noticeably improved by adding only 20 wt% ECH resin although the best compromise between compatibility and viscoelasticity corresponded to the binary blend made with 43 wt% ECH resin. The EBA copolymer + ECH resin blends showed high tack (initial adhesion) at 25 °C and some of them even at 5 °C, and they have adequate 180° peel strength both to polar (polyethylene terephthalate-PET) and nonpolar (polypropylene-PP) substrate. Furthermore, all EBA copolymer + ECH resin blends showed high shear strength at 25 °C. Finally, the blend with 43 wt% ECH resin showed excellent pressure sensitive adhesive property exhibiting excellent creep, high tack, high 180° peel strength, and high single lap-shear strength.

## 1. Introduction

Pressure sensitive adhesives (PSAs) show sufficient adhesion upon application to a substrate under light pressure during short time periods. PSAs are used as tapes and labels in electronics, food packaging, medical, and hygiene goods, among others [1]. Hot melt pressure sensitive adhesives (HMPSAs) are 100% solids that are applied in molten state on a carrier and once they cool-down to room temperature, the properties of typical PSAs are obtained. One advantage of the HMPAs with respect to the PSAs is the absence of water or organic solvents during deposition on a carrier, thereby contributing to the development of environmentally friendly adhesives [2].

The basic compositions of PSAs and HMPSAs consist of physical blends of polymer and low molecular weight resin (also named as tackifier), although small amounts (less than 10 wt%) of additives such as oils, plasticizers, stabilizers, or fillers can also be added. The thermal, surface, viscoelastic, cohesion, and adhesion properties of the PSAs and HMPSAs are related closely to the compatibility between the polymer and the resin [3]. Because the resin has lower molecular weight than the polymer, the mobility of the polymeric chains in the polymer + resin blend is favored, and, therefore, the wettability and the adhesion properties are improved. On the other hand, the resin has higher glass transition temperature (T_g_) than the polymer and, depending on the extent of compatibility, the polymer + resin blend shows intermediate T_g_ value. Therefore, the compatibility of the polymer + resin blend may determine its adhesion as PSA or HMPSA.

The PSAs based on diblock styrene-isoprene (SI) or styrene-butadiene (SB) copolymers, triblock copolymers such as styrene-isoprene-styrene (SIS) or styrene-butadiene-styrene (SBS) [4,5,6], or tetrablock copolymer [7] are the most commonly used. Although efficient, the PSAs made with styrene copolymers exhibit poor thermal, oxidative, and UV stability. Furthermore, the migration of plasticizer and low molecular weight additives to the surface occurs with time, this causes changes in their mechanical properties and adhesion. Therefore, alternate polymers are sought for obtaining PSAs with stable adhesion and improved aging resistance, the copolymers of ethylene and polypropylene have been considered very recently as potential components of HMPSAs [8,9]. The literature on HMPSAs made with polyolefin is very scarce (mainly patents) and, in this study, the use of ethylene and n-butyl acrylate (EBA) copolymer is proposed for obtaining HMPSAs with controlled viscoelastic and adhesion properties.

**Figure 1:**
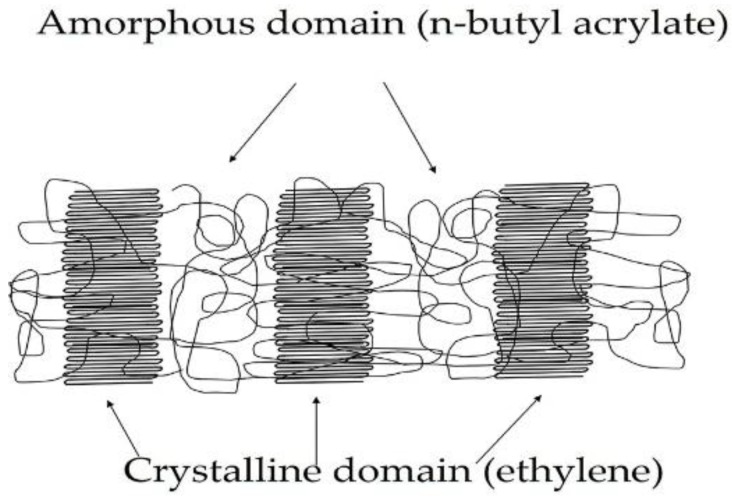
Scheme of the domains structure of the ethylene-co-n-butyl acrylate (EBA) copolymer.

EBA copolymers are potential candidates for preparing HMPSAs because of their low glass transition temperature and phase separated structure constituted by crystalline polyethylene and amorphous poly (n-butyl acrylate) domains (Figure 1). The amorphous domains impart flexibility at low temperatures to EBA copolymer and the crystalline domains impart thermal resistance and mechanical properties. However, the adhesion of EBA copolymer is poor. The adhesion of EBA copolymer can be improved by increasing its n-butyl acrylate content but the mechanical properties (i.e., cohesion) decrease, this limit its potential as adhesive. In this study, another strategy for increasing the adhesion of EBA copolymer consisting in the addition of low molecular weight resin is proposed.

There are some previous studies, patents mainly, dealing with the properties of EBA copolymer and resin blends. Brady and Kauffman [10] have prepared blends of EBA and ethylene-methyl acrylate (EMA) copolymers, polyterpene resin, and Fischer–Tropsch wax for packaging, and they have shown balanced adhesive properties. Wild [11] has synthesized EBA grafted with maleic anhydride for preparing hot melt adhesives with improved adhesion to nonpolar substrates. Liedermooy [12] has prepared blends of EBA copolymer, terpene-phenolic resin, and Fischer–Tropsch wax for improving adhesion at low temperature. Furthermore, Moyano et al. [13] have studied the rheological, thermal, and adhesion properties of blends of EBA copolymer, low molecular weight resins, and different waxes, and they found better compatibility and higher tack (i.e., immediate adhesion) for EBA-resin binary blend with microcrystalline wax compared with Fischer–Tropsch wax. The use of EBA, EVA (ethylene-co-vinyl acetate), and EMA, as well as blends of these copolymers with low density polyethylene and inorganic fillers to prepare PSA tapes, has been proposed with the aim of avoiding the plasticizer migration [14,15]. Radigon [16] has prepared blends of EBA copolymer with different co-monomer content (28–35 wt% n-butyl acrylate), melt flow indexes (MFI), and resins, and they found that the highest tack was obtained in the blends made with EBA copolymer with MFI greater than 100 g/10 min. In a recent study, Cimino et al. [8] have studied the compatibility and single lap-shear strength of EVA and EBA blends containing resins of different nature, and they have found high shear strength values and reversible adhesion in the restoration of paints.

Although several patents and a few papers have been published on PSAs made with EBA copolymer, a detailed study of the influence of the compatibility of the components of the EBA copolymer + resin blends on their adhesion properties have not been carried out yet. Furthermore, the influence of the amount of low molecular weight resin on the adhesion properties of EBA copolymer + resin blends has not been considered yet. Therefore, in this study different blends of EBA and glycerol rosin ester resin have been prepared and their potential as HMPSAs studied. Their compatibility was assessed by using different experimental techniques, and their adhesion properties to polar and nonpolar substrates were determined. The blends used in this study do not contain plasticizers or oils and they are not needed for preparing HMPSAs with outstanding properties; therefore, the properties of the HMPSAs made of EBA copolymer + resin blends will be stable over time and the migration of oils or plasticizers to the surface will not occur.

## 2. Materials and Methods

### 2.1. Materials

EBA copolymer with 27 wt% n-butyl acrylate and MFI of 150 g/10 min (Ebantix E22150, Repsol, Madrid, Spain) and hydrogenated glycerol rosin ester (ECH) resin supplied by Eastman Chemical (Kingsport, TN, USA) were used. The basic structure and some properties of the EBA copolymer and the ECH resin are given in Table 1. To prevent premature aging and to reduce the thermal degradation of the EBA copolymer + ECH resin binary blends during their preparation, 0.5 wt% Irganox1010^®^ antioxidant (BASF, Ludwigshafen, Germany) was added.

### 2.2. Preparation of the Binary Blends

A Pyrex glass crystallizer of 11.5 cm diameter was coated with a disposable aluminum container. The crystallizer was heated on a hot plate at 80 °C and the ECH resin and the antioxidant were added. Once they were melted, the temperature was increased to 160 °C and the EBA copolymer was added. Then, the crystallizer was covered with a Pyrex 3-neck glass cap, one of the connections was used for nitrogen stream (flow: 50 mL/min) to prevent oxidation, a thermometer was placed in the other connection, and one stirring rod (9 cm diameter and 3 cm height) was placed in another connection. The EBA copolymer + ECH resin + antioxidant blend was stirred with a Heidolph RZR-2000 stirrer (Heidolph Instruments, Kelheim, Germany) at 80 rpm and 160 °C for one hour. A homogeneous blend was obtained which was poured onto a Teflon^®^ sheet leaving it to solidify at room temperature.

Different blends of EBA copolymer and 20–62 wt% resin were prepared. The nomenclature of the binary blends is given in Table 2 in which the ECH resin content expressed in wt% and in phr (parts of ECH resin per 100 parts EBA copolymer) is also included.

### 2.3. Experimental Techniques

Softening point. The softening points of the EBA copolymer, ECH resin, and their binary blends were determined in a Mettler Toledo FP90 Thermosystem (Mettler Toledo GmbH, Schwerzenbach, Germany) using a FP83HT dropping point instrument with a cell having a bottom hole of 6.35 mm. The heating rate was 5 °C/min for initial survey test, and then the heating was repeated by starting at a temperature 25 °C lower than the one obtained in the survey test by using a lower heating rate (2 °C/min). Five replicates for each blend were carried out and averaged.

Differential Scanning Calorimetry (DSC). The compatibility and thermal properties of the EBA copolymer, ECH resin, and their blends were determined in a DSC TA Q100 (TA Instruments, New Castle, DE, USA) working under nitrogen atmosphere (flow: 50 mL/min). Ten milligrams of sample were placed in closed aluminum pans and the temperature was decreased to −80 °C and maintained for 3 min; then, in order to remove the thermal history, a first heating run was performed from −80 °C up to 160 °C by using a heating rate of 10 °C/min. Afterwards, the sample was cooled down from 160 °C to −80 °C with a cooling rate of 10 °C/min, and finally a second heating run was carried out from −80 °C to 160 °C with a heating rate of 10 °C/min. The crystallization of the copolymer and the binary blends was determined from the DSC cooling run and their melting and glass transition temperatures were determined from the second DSC heating run.

X-ray diffraction. The crystallinity of the EBA copolymer, ECH resin, and their binary blends were also determined in a Bruker D8-Advance diffractometer (Bruker, Ettlingen, Germany). The wavelength of copper Kα radiation (1.540598 A), copper cathode, and nickel filter with Göbel mirror were used. Scanning of 2θ angles between 5° and 90° in 0.05° steps acquired at 3 s step^−1^ was carried out.

Dynamic Mechanical Thermal Analysis (DMA). The mechanical properties of the EBA copolymer and EBA copolymer + ECH resin blends were determined using a DMA Q800 (TA Instruments, New Castle, DE, USA). Rectangular test samples with dimensions of 17 × 13 × 3 mm were prepared. Single cantilever geometry was used, the amplitude was 64 μm, the oscillation frequency was 1 Hz, and the minimum initial force was set to 1 N. A temperature scan from −80 °C up to 160 °C was carried out with a heating rate of 5 °C/min, but most of the binary blends lost their dimensional stability above 100 °C. All experiments were carried out in the region of linear viscoelasticity.

Plate–plate rheology. The viscoelastic properties of the EBA copolymer and EBA copolymer + ECH resin blends were determined in a Discovery HR-2 hybrid rheometer (TA Instruments, New Castle, DE, USA) using plate–plate geometry. Frequency sweep experiments in the region of linear viscoelasticity were carried out and the variations of the storage (G′) and loss (G″) moduli as a function of the frequency (0.1–100 rad·s^−1^) at different temperatures (20–120 °C) were recorded; a strain amplitude of 0.5% was used. According to the time–temperature superposition (TTS) principle, the master curves of the EBA copolymer + ECH resin blends were obtained; the reference temperature was 25 °C.

Transmission electron microscopy (TEM). The compatibility of the EBA copolymer + ECH resin blends was assessed by TEM micrographs obtained in a JEOL JEM-1400 Plus microscope (JEOL USA Inc., Peabody, MA, USA). Thin 100 nm thick samples were obtained in cryogenic RMC MTXL ultra microtome (Ko-Be, Japan). An acceleration voltage of 120 kV was used.

Tack. The probe tack, i.e., immediate adhesion, of the EBA copolymer, ECH resin, and their binary blends was determined in a Texture Analyzer TA.XT2i equipment (Stable Micro Systems, Surrey, UK). Cylindrical flat end steel probe of 3 mm diameter was used. The tack was measured at different temperatures controlled by means of a home-made thermostatic chamber. For measuring the tack, sample films of 200 μm thick were prepared on stainless steel plate of dimensions 60 × 60 mm by heating at 180 °C and pressing in pneumatic hot-plate press (Francisco Muñoz Irles C.B. Petrer, Spain) at 4 kg/cm^2^ for 10 s. The probe tack procedure consists in approaching the cylindrical test probe to the surface of the sample film at a rate of 0.1 mm/s, and once the probe contacted the surface of the film, a force of 5 N was applied during 1 s; then, the probe was pulled out at a rate of 10 mm/s. At least three replicates per each sample were carried out and averaged.

**Figure 2:**
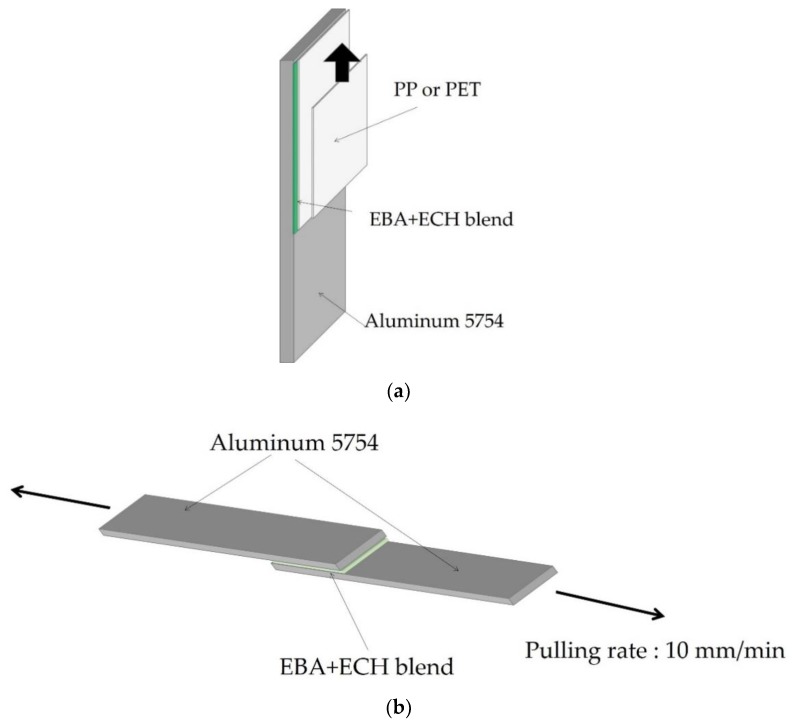
(**a**) Scheme of the test sample for 180° peel test and (**b**) scheme of the test sample for single lap-shear test.

180° peel adhesion test. The final adhesion properties of the EBA copolymer and EBA copolymer + ECH resin blends were evaluated by means of 180° peel test (Figure 2a). The substrates used were aluminum 5754 of dimensions 150 × 30 × 1.5 mm and flexible polyethylene terephthalate (PET)-polar substrate or flexible polypropylene (PP)-nonpolar substrate-film of dimensions 210 × 30 × 0.10 mm. The adhesive joints were made by placing the aluminum substrate over a heating plate at 180 °C, applying the copolymer or the binary blend over it and spreading it by means of a spatula; then, the PET or PP film was placed on the melted blend and immediately pressed in a pneumatic hot-plate press (Francisco Muñoz Irles C.B., Petrer, Spain) at 4 kg/cm^2^ for 10 s. After 1 h, the adhesive joints were tested in an Instron 4411 universal testing machine (Instron, Buckinghamshire, UK) following ASTM D 903 standard; a pulling rate of 152 mm/min was used. Five replicates for each kind of joint were tested and averaged.

Single lap-shear strength. The adhesion under shear stresses of the EBA copolymer and EBA copolymer + ECH resin blends was obtained by single lap-shear tests of aluminum 5754/copolymer or binary blend/aluminum 5754 joints (Figure 2b); aluminum 5754 test samples of dimensions 30 × 150 × 1 mm were used and the copolymer or the binary blend was applied in an area of 900 mm^2^. The copolymer or the binary blend was melted at 180 °C on the area of 900 mm^2^ of the extremes of one of the aluminum substrates, and then the other aluminum substrate was placed on top, applying a pressure of 1.1 kPa for 2 to 3 min. The joints were allowed to cool at room temperature for 2 h, and then the single lap-shear tests were carried out in an Instron 4411 universal testing machine (Instron, Buckinghamshire, UK), a cross-head speed of 10 mm/min was used. Five replicates for each kind of joint were tested and averaged.

Creep resistance under shear. Static creep shear tests of the EBA copolymer + ECH resin blends supported on PET film carrier were carried out at 24 °C and 20% relative humidity in a room temperature 10 bank shear tester (ChemInstruments, Fairfield, CT, USA) following the ASTM D3654/D3654M-02-test method A standard. A binary blend film of 20 to 40 µm thick was placed on an area of 2.4 × 2.4 cm^2^ of PET film of dimensions 25.4 × 180 mm which was placed onto polished stainless steel 304 passing a 2 kg rubber coated roller 30 times over the joint. Then, the samples were placed into the creep shear tester and a standard load of 1 kg was hanged. The time required by the binary blend/PET piece to fall was measured (holding time) and it was taken as the measurement of the static creep shear strength. Three replicates for each binary blend supported on PET film carrier were carried out and averaged.

## 3. Results and Discussion

### 3.1. Compatibility of the EBA Copolymer + ECH Resin Blends

The compatibility of the EBA copolymer and the low molecular weight ECH resin determines the phase structure, physical properties, and adhesion properties of the binary blends [17]. Because the EBA copolymer contains polar acrylate and nonpolar ethylene moieties, a low molecular weight resin containing both polar ester and nonpolar cycloaliphatic moieties was selected in this study. Several experimental techniques were used for assessing the compatibility of the EBA copolymer + ECH resin blends.

**Figure 3:**
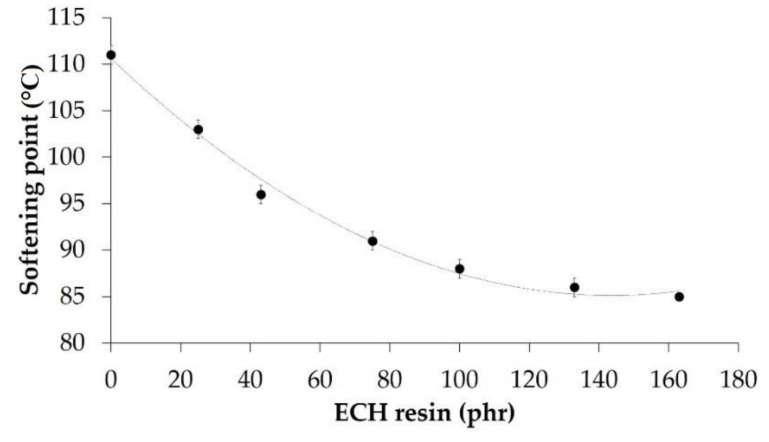
Variation of the softening point of the EBA copolymer + ECH resin blends as a function of their ECH resin content. Softening point of the ECH resin: 103 °C.

It has been established [18] that the chemical potential of a crystallizable polymer decreases when adding a miscible diluent resulting in decreased melting point. Furthermore, the softening point test has been proposed as a method to find the phase behavior of amorphous polymer blends, a decrease of the softening temperature in compatible polymer blends was evidenced [19]. Therefore, the lower softening point of a binary blend with respect to the softening points of the parent components is an indication of the miscibility or compatibility of the blend. Figure 3 shows the variation of the softening point of the EBA copolymer + ECH resin blends as a function of their ECH resin content. The addition of a small amount of ECH resin decreases the softening point of the EBA copolymer, more markedly by adding up to 50 wt% (100 phr) ECH resin, indicating the existence of compatibility. The softening points of all EBA copolymer + ECH resin blends are lower than the one of the ECH resin, and the addition of more than 50 wt% ECH does not change the softening point of the binary blends.

**Figure 4:**
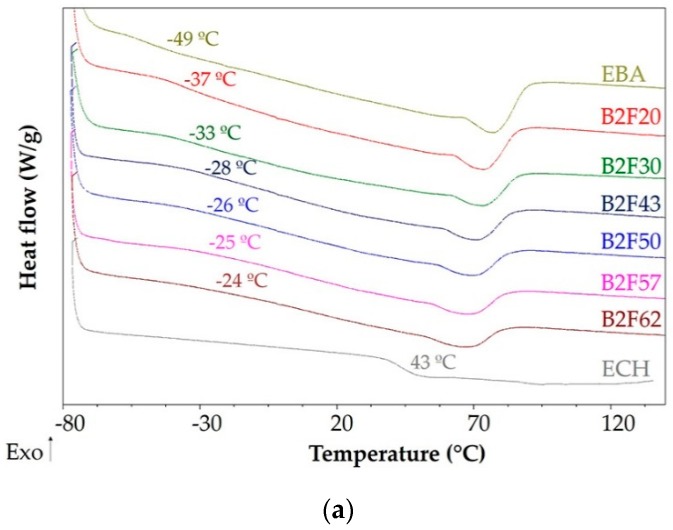

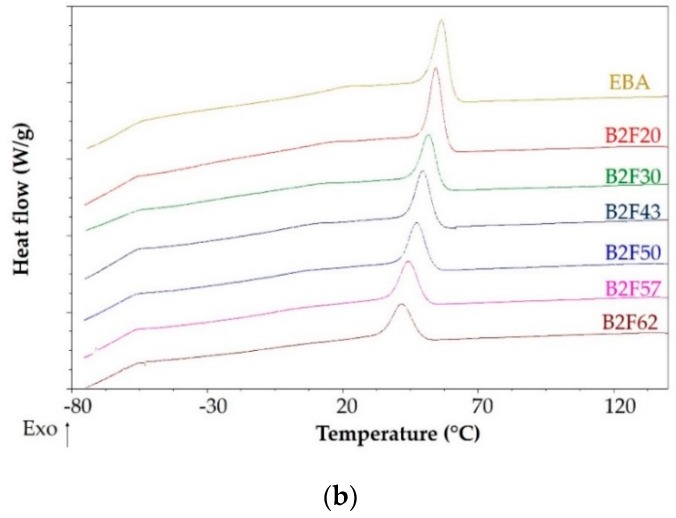
(**a**) Differential Scanning Calorimetry (DSC) thermograms of the EBA copolymer, ECH resin, and their binary blends. Second heating run; (**b**) DSC thermograms of the EBA copolymer, ECH resin, and their binary blends: Cooling run.

DSC experiments were also carried out to evaluate the miscibility between the EBA copolymer and the ECH resin in the blends. Figure 4a shows the DSC thermograms of the EBA copolymer, the ECH resin and their binary blends. The glass transition (T_g_) and melting (T_m_) temperatures of the EBA copolymer + ECH resin blends were evaluated from the second DSC heating run, and the glass transition can be observed as an inflexion; the melting temperature is taken as the minimum of the endothermic peak. The T_g_ value of the EBA copolymer is −49 °C and the one of the ECH resin is 43 °C. The presence of multiple glass transition temperatures can be used as a criterion of incompatibility in binary blends. However, because the T_g_ value of the polyethylene domain in EBA is the only one observed in the DSC thermograms of the binary blends, their compatibility from the T_g_ values of the blends cannot be assessed properly. However, an increase in the T_g_ value of the polyethylene domain in EBA copolymer by increasing the amount of ECH resin up to 50 wt% is observed, suggesting an increase of the compatibility; nevertheless, the binary blends containing more than 50 wt% ECH resin show similar compatibility (Figure 4a). These results agree well with the ones obtained by measuring the softening points of the binary blends.

A blend of two fully compatible polymers with different T_g_ values will exhibit one single T_g_, which value will depend on the T_g_ values of each polymer and their weight fractions in the binary blend [20]. The T_g_ value of a fully compatible blend can be determined by means of the Fox equation:

1/T_g_ = w_1_/T_g1_ + w_2_/T_g2_ (1)

where T_g1_ and T_g2_ are the glass transition temperatures and w_1_ and w_2_ are the weight fractions of the two components of the blend. The T_g_ values of the EBA copolymer + ECH resin blends obtained by applying the Fox equation are given in Table 3. Whereas, the experimental and theoretical T_g_ values of the binary blends containing up to 30 wt% ECH resin are somewhat similar indicating very good compatibility, the experimental T_g_ values of the blends with 43 wt% or more ECH resin are lower than the T_g_ values predicted by Fox equation, the differences are more noticeable when increasing the resin content, indicating partial compatibility of the EBA copolymer and the ECH resin.

The compatibility of the EBA copolymer + ECH resin blends should produce a change in the domain structure of the EBA copolymer. In fact, the DSC thermogram of the EBA copolymer of Figure 4a shows the melting of the polyethylene domains of the copolymer at 78 °C. The addition of ECH resin decreases the melting temperature (T_m_) and enthalpy (ΔH_m_) of the EBA copolymer, more markedly when adding up to 50 wt% ECH (Table 3), indicating that the resin is decreasing the interactions between the ethylene chains in the polyethylene domains. In fact, Figure 4b shows the crystallization peak of the polyethylene domains of EBA in the cooling DSC run at 56 °C, and the addition of ECH resin decreases the temperature (T_c_) and enthalpy (ΔH_c_) of crystallization of the EBA copolymer (Table 3), confirming the disruption of the interactions between the polyethylene domains. The decrease in the crystallinity of the EBA copolymer is more marked when increasing the ECH resin content in the binary blend up to 50 wt%, and similar crystallinity is obtained in the blends containing more than 50 wt% ECH resin. The crystallinity (X_c_) of the EBA copolymer + ECH resin blends can be also calculated from their melting enthalpies by using Equation (2):

(2) $$\mathrm{Xc}=\frac{\Delta H_{f}}{\Delta H_{f}^{*}}\times 100\%$$

where $\Delta H_{f}^{*}$ is the melting enthalpy of completely crystalline polyethylene (277.1 J/g [21]) and ΔH_f_ is the melting enthalpy of the binary blend. The crystallinity (X_c_) of the EBA copolymer is 7.9% and decreases to 2.1% in the binary blend containing 43 wt% ECH resin and to 1.1% in the blend with 62 wt% ECH resin (Table 3). In summary, the addition of the ECH resin to EBA copolymer increases the compatibility and reduces the crystallinity of the polyethylene domains.

**Figure 5:**
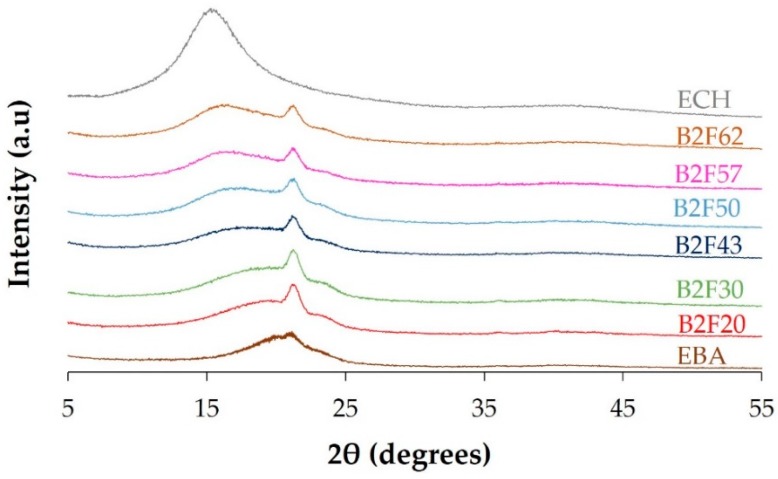
X-ray diffractograms of the EBA copolymer, the ECH resin and their binary blends.

**Figure 6:**
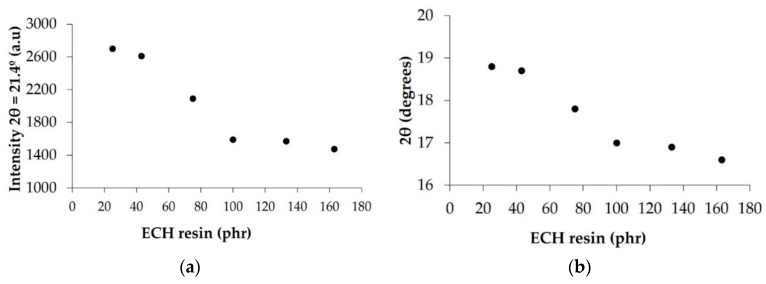
(**a**) Variation of the intensity of the diffraction peak at 2θ = 21.4 degrees of the EBA copolymer + ECH resin blends as a function of their ECH resin content. X-ray diffraction experiments. (**b**) Variation of the 2θ value of the amorphous part of the EBA copolymer + ECH resin blends as a function of their ECH resin content. X-ray diffraction experiments.

The crystallinity of EBA copolymer + ECH resin blends was also determined by X-ray diffraction experiments. The X-ray diffractograms of the EBA copolymer, the ECH resin, and their binary blends are shown in Figure 5. The X-ray diffractogram of the EBA copolymer shows a broad peak between 15 and 25 degrees and a slight diffraction peak at 2Θ = 21.4 degrees (associated to the orthorhombic phase of the polyethylene domains in the EBA copolymer [22]). On the other hand, the X-ray diffractogram of the ECH resin shows a broad peak between 10 and 20 degrees, indicating that it is amorphous. The addition of the ECH resin broadens the amorphous diffraction peak (due to the poly(n-butyl acrylate) domains) of the EBA copolymer in the blends and the narrow diffraction peak of the polyethylene domains at 2Θ = 21.4 degrees is more clearly distinguished, pointing to higher degree of separation between the amorphous and crystalline domains in the blends. Due to higher degree of phase separation between the polyethylene and poly(n-butyl acrylate) domains of the EBA copolymer, the diffraction peak of the polyethylene domain is better distinguished in the blends than in the copolymer. The intensity of the diffraction peak of the crystalline part of the binary blends at 2Θ = 21.4 degrees decreases when increasing the amount of ECH resin up to 50 wt% (Figure 6a), this indicates a decrease of the crystallinity, and the intensity does not vary in the blends containing more than 50 wt% (100 phr) ECH resin; these trends agree well with the evidences provided by the DSC experiments. On the other hand, the maximum of the broad diffraction peak of the amorphous part of the binary blends decreases from 2Θ = 18.8 degrees in B2F20 blend to 2Θ = 16.6 degrees in the B2F62 blend, likely due to the miscibility between the polar ester moieties of the ECH resin and the polar acrylate groups of the EBA copolymer. In fact, Figure 6b shows a decrease in 2Θ value of the peak of the amorphous part of the binary blends by increasing their ECH resin content up to 50 wt%, the 2Θ value does not vary in the binary blends containing 50 wt% or more ECH resin.

**Figure 7:**
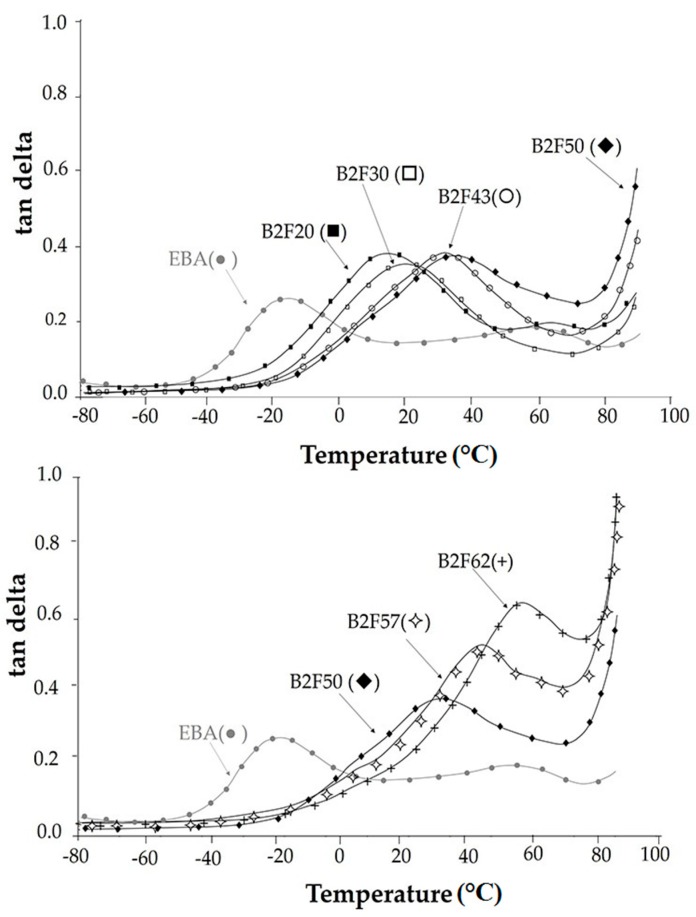
Variation of tan delta as a function of the temperature for EBA copolymer and EBA copolymer + ECH resin blends. Dynamic mechanical thermal analysis (DMA) experiments.

DMA has been demonstrated to be useful [13] for analyzing the compatibility of the EBA copolymer + resin blends. Figure 7 shows the variation of tan delta (=E″/E′) as a function of the temperature for the EBA copolymer and EBA copolymer + ECH resin blends. For ethylene-based copolymers three structural relaxations named α, β, and γ have been observed between −140 °C and 60 °C [23,24]. The α structural relaxation transition appears at high temperature and is associated with the movements of large sections of the main polymeric chains once the crystallites begin to melt. β structural relaxation occurs at intermediate temperature and is attributed to segmental motions of disordered chains, occurring within the interfacial regions associated to the lamellar crystallites. The γ structural relaxation appears at low temperature (near −130 °C) and corresponds to the glass transition of the polyethylene. In this study, only the α and β structural relaxations are evidenced in the tan delta vs. temperature plots. Most blends show only β structural relaxation, but the EBA copolymer and the blend containing 20 wt% ECH resin show both α and β structural relaxations (Table 4); the α structural relaxation cannot be distinguished in the blends with 30 wt% or more ECH resin because it appears in the melting region of the DMA curves. Figure 7 shows that the addition of the ECH resin shifts the β structural relaxation of the EBA copolymer to higher temperature more markedly when increasing the ECH resin content, indicating the compatibility of the blends containing up to 50 wt% ECH resin; furthermore, the addition of the ECH resin increases the value of tan delta in the maximum due to the decrease of the crystallinity of the EBA copolymer. However, the addition of higher amounts of ECH resin does not change the value of tan delta in the maximum (Figure 7, Table 4) because of the spatial distribution of the two phases (phase enriched in ECH resin and enriched in EBA) is not changing noticeably due to the interfacial interactions between both phases [25]. On the other hand, the β structural relaxation of the blends containing more than 50 wt% ECH resin is displaced to higher temperature and the value of tan delta in the maximum increases (Figure 7) due to an important decrease of the crystallinity and the dominance of the ECH resin domains in the structure.

**Figure 8:**
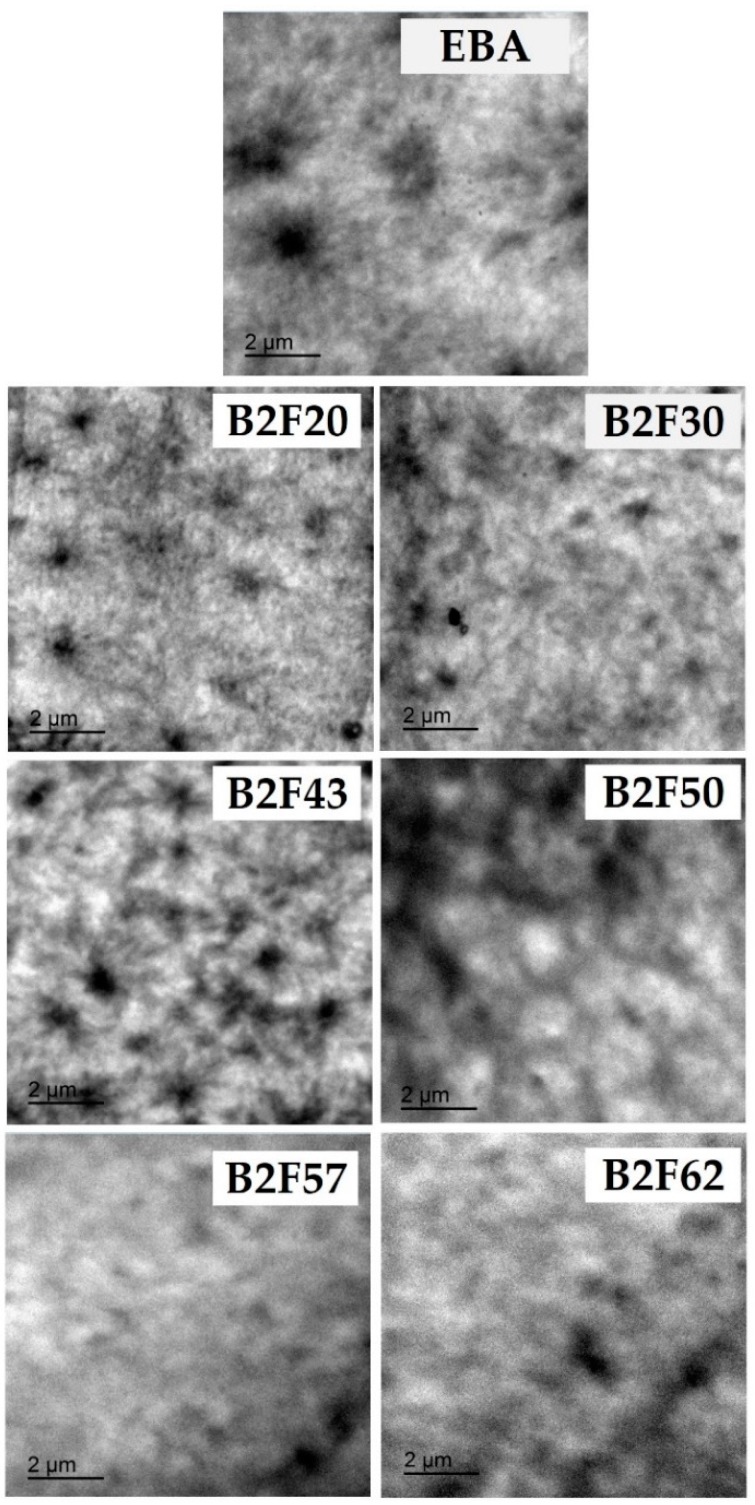
TEM micrographs of the EBA copolymer and EBA copolymer + ECH resin blends.

The compatibility of the EBA copolymer + ECH blends was also studied by TEM. The TEM micrographs of the EBA copolymer and the binary blends are shown in Figure 8. The TEM micrograph of the EBA copolymer shows two phases due to polyethylene and poly(n-butyl acrylate) domains, and the addition of up to 50 wt% ECH resin decreases the size of the polyethylene domains (from 1 μm in the EBA copolymer to 0.4–0.6 μm in the blends), confirming the decrease in the crystallinity and the improved compatibility in the blends, in agreement with the results of the softening point measurements, DSC, X-ray diffraction, and DMA. On the other hand, when the amount of ECH resin in the binary blends is higher than 50 wt%, the existence of the two phases is not so clear, because the ECH resin enriched phase is dominant.

In summary, the experimental results obtained by the measurement of the softening points, the X-ray diffractograms, the DSC thermograms, the DMA curves, and the TEM micrographs of the EBA copolymer + ECH resin blends show two different compatibility behaviors. For the binary blends containing up to 50 wt% ECH resin, the compatibility increases by increasing the ECH resin content, whereas for those blends containing more than 50 wt% ECH resin the compatibility is similar irrespective of the resin content. These two different compatibility behaviors should affect differently to the viscoelastic and the adhesion properties of the binary blends.

### 3.2. Viscoelastic Properties of the EBA Copolymer + ECH Resin Blends

Pressure sensitive adhesives should have an adequate viscoelasticity, i.e., they must satisfy the Dahlquist criterion for exhibiting tack at room temperature and the elastic or storage modulus must be lower than 3.5 × 10^5^ Pa [26]. Furthermore, a pressure sensitive adhesive must be able to wet a substrate upon application of very light pressure but it should be separated by applying a moderate stress, i.e., a good balance between adhesion and cohesion must be obtained [27].

**Figure 9:**
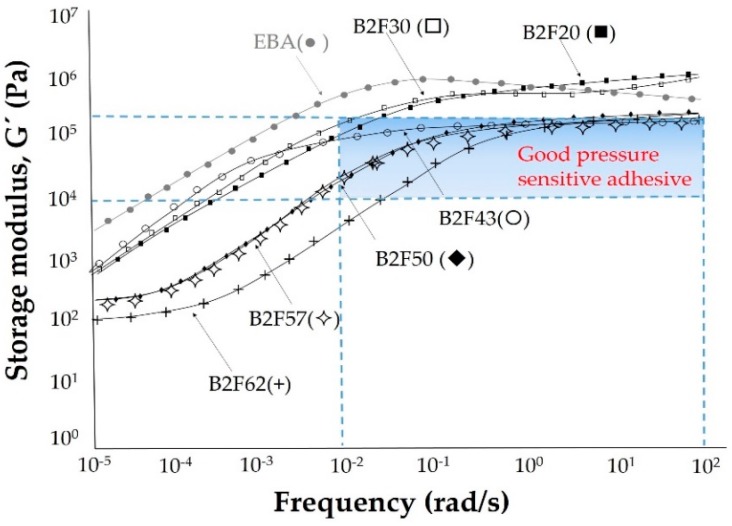
Variation of the storage modulus (G′) as a function of the frequency for the EBA copolymer and EBA copolymer + ECH resin blends. Plate–plate rheology. Frequency sweep. T_ref_ = 25 °C.

The viscoelastic properties of the EBA copolymer + ECH resin blends were determined by frequency sweep plate–plate rheology experiment and the variation of the storage modulus (G′) as a function of the frequency is shown in Figure 9. At high frequency, in the glassy region, the storage modulus decreases in the binary blends containing 43 wt% or more ECH resin, likely due to the decrease in the crystallinity of the EBA copolymer. When the glass transition is produced, the storage moduli of the blends decrease with decreasing frequency. For the binary blends both the storage modulus and the frequency at which the glass transition starts decrease when increasing their ECH resin content, this indicates the miscibility between the EBA copolymer and the ECH resin. On the other hand, the viscoelastic curves of the B2F20 and B2F30 blends are very similar but the one for B2F43 shows lower storage modulus at high frequency, the beginning of the glass transition is displaced to high temperature and the storage modulus is higher at low frequencies. Therefore, the viscoelastic properties of the B2F43 blend are somewhat “anomalous”. The lowest storage moduli correspond to the binary blends containing 50 wt% or more ECH resin.

**Figure 10:**
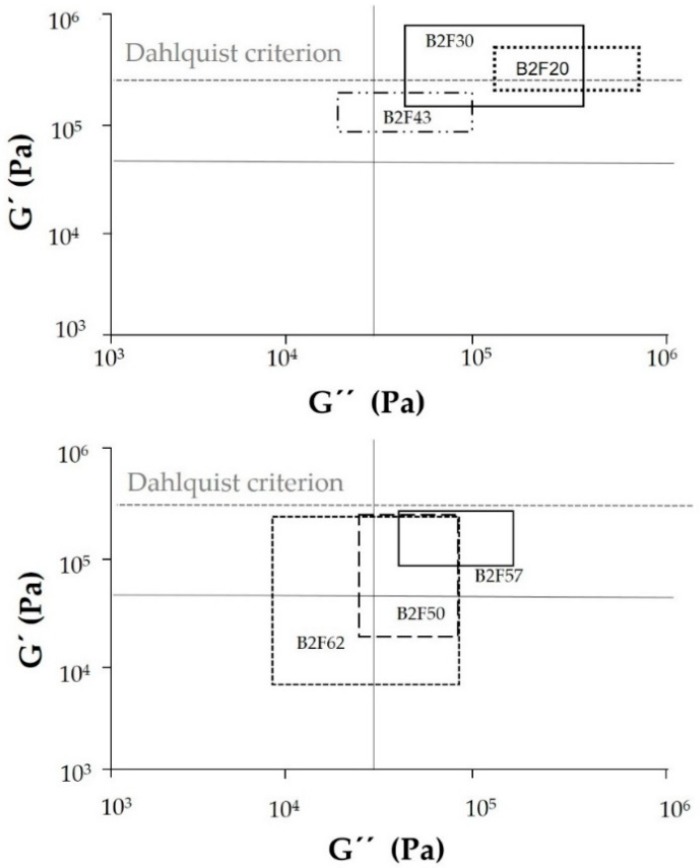
Chang’s viscoelastic windows for EBA copolymer + ECH resin blends.

Figure 9 shows that the Dalhquist criterion is obeyed by the binary blends containing 43 wt% or more ECH resin indicating that these blends show good pressure sensitive adhesive property. Furthermore, the optimal range of the storage moduli for pressure sensitive adhesive property is from 10^4^ to 10^5^ Pa in the frequency range of 0.01 to 100 rad/s [28], and the binary blends containing 43 wt% or more ECH resin also show their viscoelastic properties in this range. In the early 1990s, Chang developed a theory to interpret the rheological data of pressure sensitive adhesives and established criteria for their classification when used in conjunction with the Dahlquist criterion [26]. The Chang viscoelastic windows can be obtained by plotting the values of G′ vs. G″ at frequencies of 0.01 rad/s and 100 rad/s which are associated with the bonding and debonding processes of the pressure sensitive adhesives, respectively, and the four quadrants corresponding to high-shear, removable, low-temperature, and general purpose pressures sensitive adhesives can be obtained. Furthermore, Chang established that for the most PSAs, the range of G’ values at room temperature at frequencies of 0.01 rad/s and 100 rad/s was l0^3^ to 10^6^ Pa. Figure 10 shows the Chang viscoelastic windows of the EBA copolymer + ECH resin blends. Whereas all blends have bonding moduli (i.e., the base of the window) below the Dahlquist criterion (which means good conformability), the debonding moduli (i.e., the upper part of the window) are below the Dahlquist criterion for the binary blends containing 43 wt% or more ECH resin. The binary blends with lower ECH resin content can be considered high shear PSAs and when increasing the ECH resin content, they become general pressure sensitive adhesives (B2F62). Therefore, by changing the ECH resin content in the binary blends, pressure sensitive adhesives with different performance properties can be obtained.

### 3.3. Adhesion Properties of the EBA Copolymer + ECH Resin Blends

**Figure 11:**
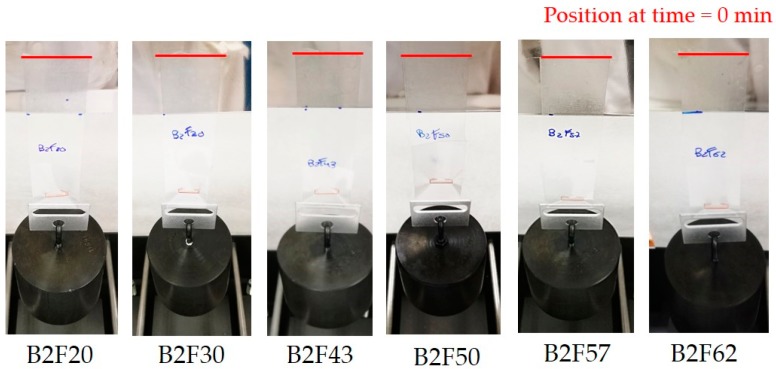
Creep resistance of the EBA copolymer + ECH resin blends after three days.

The pressure sensitive adhesives must show a good balance between cohesion and adhesion. The cohesion of the EBA copolymer + ECH resin blends was estimated from the holding time in creep experiments under shear. Figure 11 shows the aspect of the different binary blends applied on PET film after three days of being placed in the equipment for measuring creep. All binary blends show the same creep resistance and none of them fall after three days nor slide from the polished stainless steel plate. Therefore, all binary blends show excellent cohesion.

The adhesion properties of the EBA copolymer + ECH resin blends were determined by measuring the tack, the 180° peel strength, and the single lap-shear strength.

**Figure 12:**
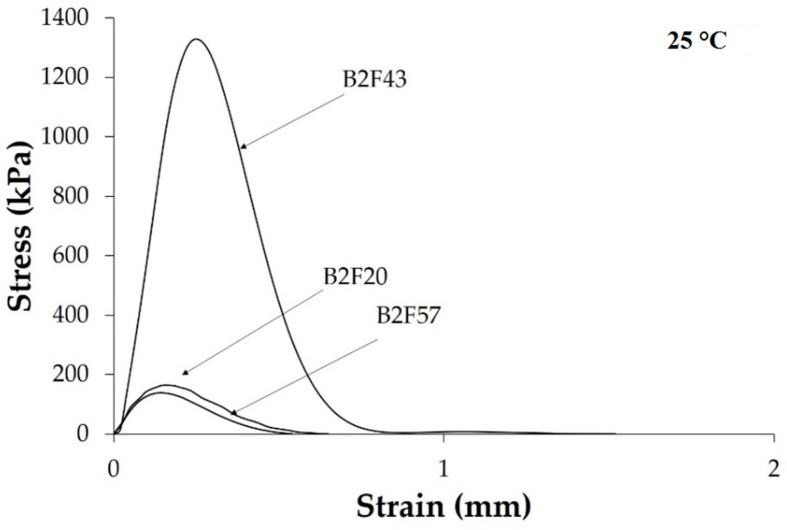
Stress vs. strain curves at 25 °C of some EBA copolymer + ECH resin blends.

The tack of the EBA copolymer + ECH resin blends was determined using the probe tack method. In this method, the stress–strain curves are obtained and the maximum of the curve is taken as the tack of the binary blend. In a previous study [29] it has been proposed that the shapes of the stress–strain curves of the pressure sensitive adhesives can be related to their viscoelastic response. Thus, the viscoelastic response of a solid pressure sensitive adhesive (i.e., the viscoelastic elastic regime is dominant) corresponds to a stress–strain curve having maximum stress at low strain values, thus the area under the curve is small, whereas the viscoelastic response of a liquid pressure sensitive adhesive (i.e., the viscous viscoelastic regime is dominant) corresponds to a stress–strain curve having maximum stress at medium–high strain values, thus the area under the curve is large. The stress–strain curves at 25 °C of the EBA copolymer + ECH resin blends are given in Figure 12. The stress–strain curves at 25 °C of all binary blends exhibit a maximum at moderate strain, indicating that they have solid pressure sensitive adhesive behavior and, consequently, an interfacial failure between the probe and the surface of the blend is produced during the tests, i.e., the formation of fibrils is not important.

The values of the tack at 25 °C of the EBA copolymer + ECH resin blends obtained from the maximum of the stress–strain curves are given in Table 5. The tack of the binary blend increases by increasing the amount of ECH resin up to 43 wt% decreasing for higher ECH resin loading. This trend is in agreement with the two compatibility behaviors defined above, i.e., the increase of the compatibility of the binary blends containing up to 50 wt% ECH resin and the lack of variation of the compatibility in the binary blends with 50 wt% or more ECH resin. The tack at 25 °C is extremely high for B2F43 likely due to its particular viscoelastic behavior (Figure 9), good compatibility, and phase domain morphology. On the other hand, the areas under the stress–strain curves are related to the work of adhesion developed during debonding at 25 °C. A similar trend than the one for the tack at 25 °C is obtained (Table 5), indicating good performance of the B2F43 blend as a pressure sensitive adhesive.

**Figure 13:**
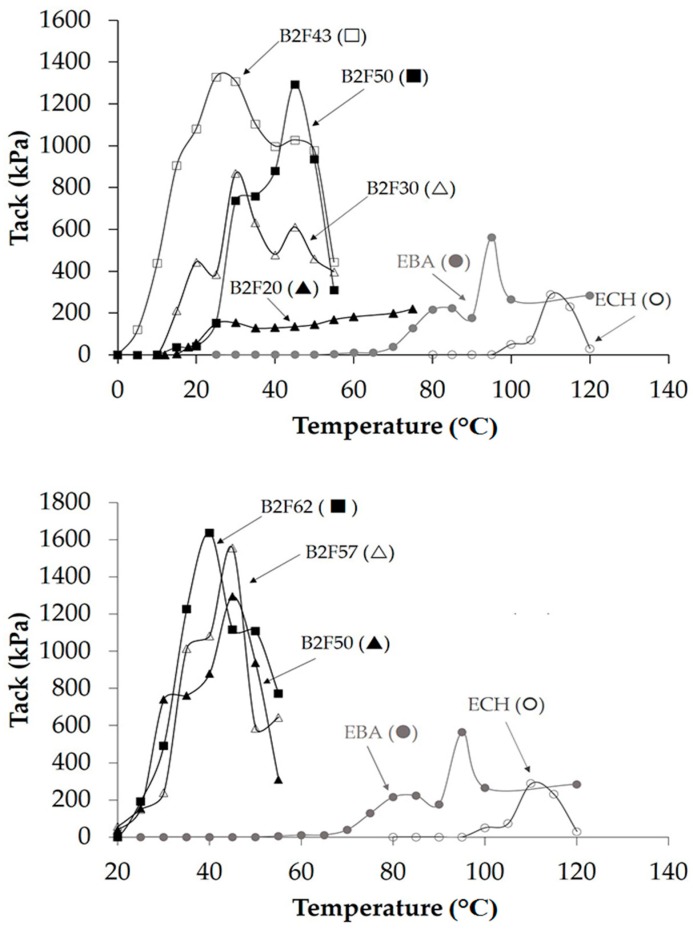
Variation of the tack of the EBA copolymer, ECH resin, and their binary blends as a function of the temperature.

The tack of the EBA copolymer + ECH resin blends was measured at different temperatures and the variation of the tack as a function of the temperature is given in Figure 13. Whereas the ECH resin exhibits low tack above 100 °C and the EBA copolymer shows reasonable tack at about 70 °C, all binary blends are tacky at temperatures lower than 60 °C and the tack is higher than for the individual components of the blends (B2F20 is an exception) because of good compatibility. In fact, it has been shown that the better compatibility between EVA copolymer and resin produced an improvement of the adhesion properties of the EVA copolymer + resin blends [30]. The increase of the ECH resin content up to 43 wt% displaces the maximum of the tack of the binary blend to lower temperature, and the addition of more than 43 wt% ECH resin shifts the maximum of the tack to higher temperature (Figure 13, Table 5); this trend agrees well with the two compatibility behaviors in the binary blends shown above. On the other hand, Table 5 shows that the tack of the binary blends increases by increasing their ECH resin content, and the range of temperatures with tack is, in general, between 20 and 55 °C. Therefore, the compatibility determines the tack of the binary blends but it is also influenced by their degree of crystallinity and their viscoelastic properties.

**Figure 14:**
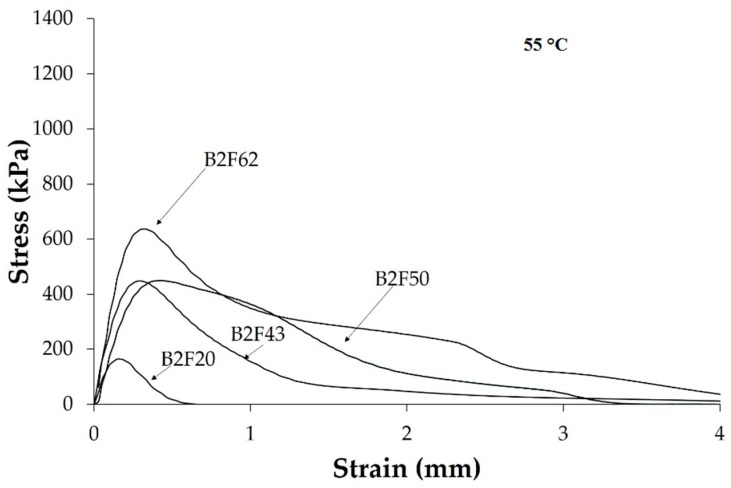
Stress vs. strain curves at 55 °C of some EBA copolymer + ECH resin blends.

Because the maximum tack of the most binary blends is extended above 25 °C, the stress–strain curves at 55 °C of the EBA copolymer + ECH resin blends were also studied (Figure 14). At 55 °C the tack of the binary blends increases when increasing their ECH resin content and fibrillation appears in all blends containing 43 wt% or more ECH resin, the extent of fibrillation increases when increasing the ECH resin content. This trend can be ascribed to the dominant viscous regime of the blends with 43 wt% or more ECH resin at 55 °C, the pressure sensitive adhesive corresponds to that of liquid state one [29].

**Figure 15:**
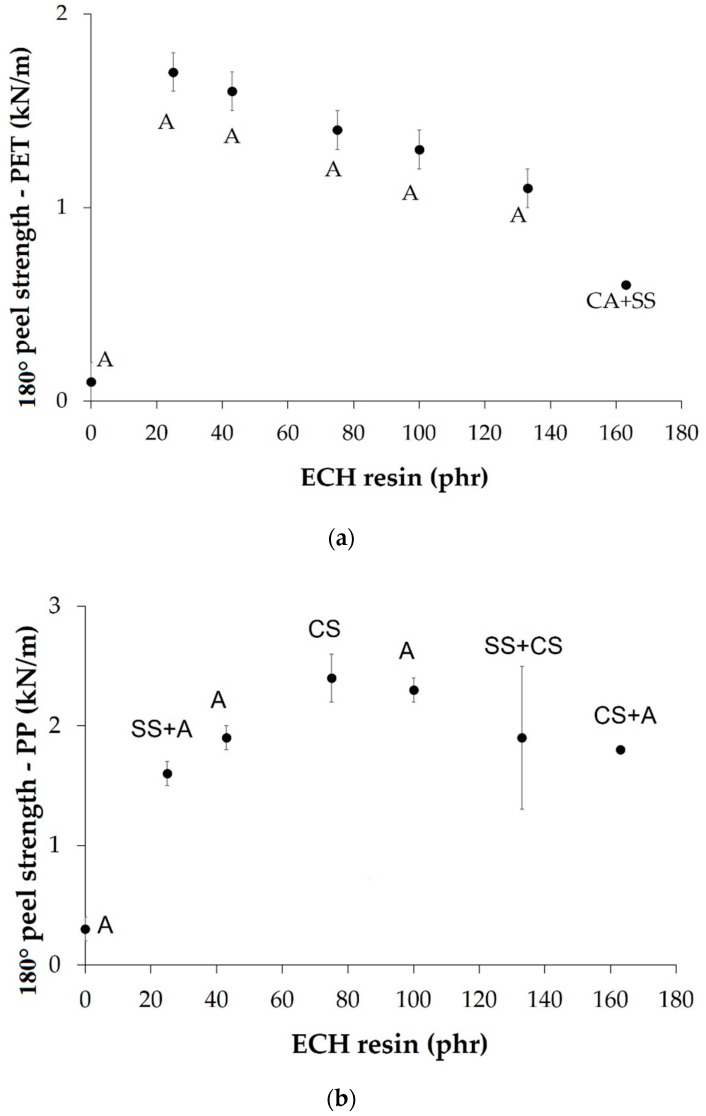
(**a**) Variation of the 180° peel strength of aluminum/EBA copolymer or EBA copolymer + ECH resin blend/PET film joints as a function of the amount of ECH in the binary blend. Locus of failure: A: Adhesive failure to PET film, CA: Cohesive failure of the adhesive, SS: Stick-slip. (**b**) Variation of the 180° peel strength of aluminum/EBA copolymer or EBA copolymer + ECH resin blend/PP film joints as a function of the amount of ECH in the binary blend. Locus of failure: A: Adhesive failure to PP film, CA: Cohesive failure of the adhesive, SS: Stick-slip, CS: Cohesive failure of PP film.

Because the EBA copolymer contains polar poly(n-butyl acrylate) and nonpolar polyethylene domains, adhesion to both polar and nonpolar substrates is feasible. Therefore, the adhesion under peel stresses of the EBA copolymer + ECH resin blends has been measured in joints made with polar (PET film) and nonpolar (PP film) substrates. Figure 15a shows the variation in the 180° peel strength of aluminum/EBA copolymer or binary blend/PET film joints as a function of the amount of ECH resin in the binary blend. The 180° peel strength of the joint made with EBA copolymer is very low (0.1 kN/m), and the addition of ECH resin results in an increase in the 180° peel strength; the highest 180° peel strength (1.7 kN/m) corresponds to the joint made with the binary blend containing 20 wt% (25 phr) ECH resin, and decreases steadily by increasing the amount of ECH resin in the binary blend. The most joints show an adhesive failure to the PET film because of higher adhesion to aluminum substrate, and the joint made with B2F62 shows a mixed failure of stick-slip and cohesive failure in the adhesive. The compatibility of the binary blend favors an increase in the 180° peel strength of the joints to PET film but the better compatibility of the binary blend does not improve the 180° peel strength indicating that other factors, such as the surface energy and the interfacial adhesion, determine the adhesion of the binary blends to PET films.

Figure 15b shows the variation in 180° peel strength of the aluminum/EBA copolymer or binary blend/PP film joints as a function of the amount of ECH resin in the binary blend. The 180° peel strength of the joint made with EBA copolymer is very low (0.3 kN/m), and the addition of ECH resin results in an increase of the 180º peel strength, the value obtained is quite high (2.4 kN/m). The 180º peel strength value increases by increasing the ECH resin content of the binary blend up to 50 wt%, decreasing slightly in the joints made with the binary blends containing 50 wt% (100 phr) or more ECH resin. This trend agrees well with the trend in the compatibility of the EBA copolymer+ECH resin blends and with the existence of two different compatibility behaviors. The loci of failure of the joints vary widely from adhesive failure to PP film to cohesive failure of the PP film, this last one confirm the excellent adhesion of the EBA copolymer + ECH resin blends to PP film. The different loci of failure of the joints can be ascribed to the different mechanical properties of the blends which decrease when increasing their ECH resin content (Figure 9). Thus, the joints made with binary blends containing 50 wt% or more ECH resin have lower storage moduli and, therefore, the resistance to peel stresses decrease and mixed loci of failure with contributions of the stick-slip and the adhesive failure are obtained.

**Figure 16:**
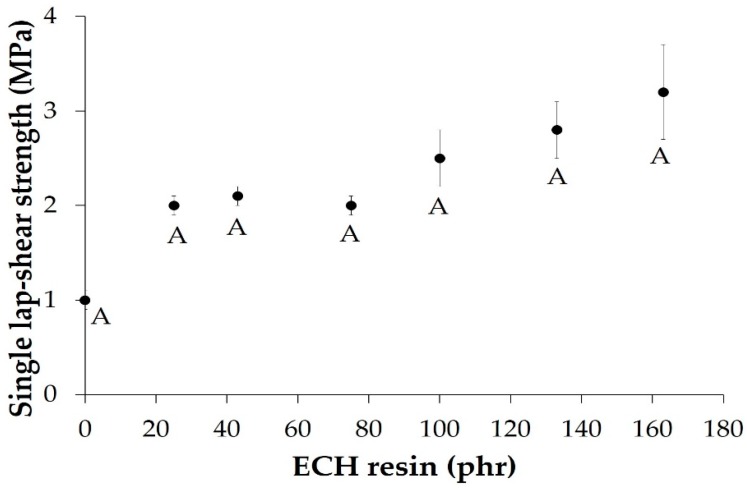
Variation of the single lap-shear strength of aluminum/EBA copolymer + ECH resin blend/aluminum joints as a function of the amount of ECH in the binary blend. Locus of failure: A: Adhesive failure.

Finally, the variation of the single lap-shear strength of aluminum/EBA copolymer or binary blend/aluminum joints as a function of the amount of ECH resin in the blend is shown in Figure 16. Because the adhesion is measured under shear stresses, the lap-shear strength of the joint made with the EBA copolymer is relatively high (1 MPa) because of its high storage modulus (Figure 9). The lap-shear strength increases more than two-fold in the joint made with the binary blend containing 20 wt% (25 phr) ECH resin, and the lap-shear strength does not vary by increasing the ECH resin content up to 50 wt% (100 phr), increasing more noticeably in the joints made with the binary blends containing more than 50 wt% ECH resin. A value of lap-shear strength higher than 3 MPa is obtained in the joint made with B2F62 blend, the maximum value obtained for B2F43 being quite high for a physical blend. Again, the single lap-shear adhesion of the EBA copolymer + ECH resin blends is governed by their compatibility. An adhesive failure was always obtained.

## 4. Conclusions

The addition of hydrogenated glycerol rosin ester resin (ECH) improved the compatibility of the EBA copolymer and decreased the crystallinity and size of the polyethylene domains. Depending on the amount of ECH resin added, two different compatibility behaviors in the binary blends were obtained. The addition of amounts of ECH resin up to 50 wt% (100 phr) improved the compatibility, whereas the addition of more than 50 wt% ECH resin did not change the compatibility of the binary blend but the viscoelastic properties were noticeably decreased. Furthermore, the compatibility of the binary blends was noticeably improved by adding only 20 wt% (25 phr) ECH resin although the best compromise between compatibility and viscoelastic properties corresponded to the binary blend made with 43 wt% ECH resin.

The tack and adhesion properties of the EBA copolymer + ECH resin blends were determined by their compatibility and viscoelastic properties. The tack of the binary blends was higher than the one of the EBA copolymer and the ECH resin, and the tack appeared at lower temperatures. The tack at 25 °C and the work of adhesion of the binary blends increased when increasing their ECH resin content up to 43 to 50 wt% (75–100 phr) and decreased for the binary blends containing more than 50 wt% ECH resin. However, the maximum tack increased by increasing the ECH resin content in the binary blends because of the contribution of the viscous component, although the maximum tack appeared at temperatures above 40 °C in the binary blends containing 50 wt% (100 phr) or more ECH resin. All binary blends had good cohesion and excellent creep resistance. On the other hand, the trends in the 180° peel strength to PP film and the single lap-shear strength were similar to the one in the compatibility confirming that the compatibility affected the adhesion of the binary blends. The 180° peel strength and the single lap-shear strength obtained with the joints made with the binary blends are quite high as compared to the most of the pressure sensitive adhesives. Finally, the EBA copolymer + ECH resin blends showed high-shear and are general purpose pressure sensitive adhesives, the best balance in properties was found in the binary blend containing 43 wt% ECH resin.

## Figures and Tables

**Table 1:** Nomenclature, chemical structure, and some characteristics of the raw materials.

Raw Material	Structure	Property
**EBA**	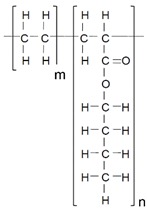	MFI = 150 g/10 min (190 °C; 2.16 kg)
		n-butyl acrylate = 27 wt%
		Density (23 °C) = 0.925 g/cm^3^
		Melting point = 78 °C
		Glass transition temperature (T_g_) = −49 °C
**ECH resin**	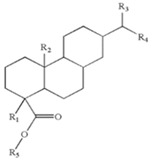	Softening point = 103 °C
		Glass transition temperature (T_g_) = 43 °C
		R_1_, R_2_, R_3_, R_4_, R_5_: Short alkyl chains

**Table 2:** Composition of the EBA copolymer + hydrogenated glycerol rosin ester (ECH) resin binary blends.

Nomenclature	Composition (wt%)	ECH Amount	
		phr *	Weight (wt%)
B2F20	80% EBA + 20% ECH	25	20
B2F30	70% EBA + 30% ECH	43	30
B2F43	57% EBA + 43% ECH	75	43
B2F50	50% EBA + 50% ECH	100	50
B2F57	43% EBA + 57% ECH	133	57
B2F62	38% EBA + 62% ECH	163	62

(*) phr: part of ECH resin per 100 parts EBA copolymer.

**Table 3:** Some parameters obtained from DSC thermograms of the EBA copolymer, the ECH resin, and their binary blends.

Blend	T_g_ (°C)	T_g_ Fox Eq (°C)	T_m_ (°C)	ΔH_m_ (J/g)	X_c_ (%)	T_c_ (°C)	ΔH_c_ (J/g)
EBA	−49	-	78	8	7.9	56	20
B2F20	−37	−35	75	7	2.7	54	17
B2F30	−33	−28	74	6	2.4	52	14
B2F43	−28	−17	73	6	2.1	49	14
B2F50	−26	−11	72	5	1.8	47	14
B2F57	−25	−4	71	4	1.4	44	13
B2F62	−24	0	71	3	1.1	42	12
ECH	43	-	-	-	-	-	-

**Table 4:** Values of maximum tan delta and temperature of the structural relaxations in the EBA copolymer and EBA copolymer + ECH resin blends. DMA experiments.

Blend	T_β_ (°C)	Tan Delta_β_	T_α_ (°C)	Tan Delta_α_
EBA	−18	0.25	50	0.20
B2F20	9	0.37	60	0.20
B2F30	16	0.35	-	-
B2F43	27	0.38	-	-
B2F50	30	0.37	-	-
B2F57	41	0.52	-	-
B2F62	53	0.64	-	-

**Table 5:** Values of maximum tack, temperature of maximum tack, range of temperature with tack, tack at 25 °C, and work of adhesion for EBA copolymer and EBA copolymer + ECH resin blends.

Blend	Max Tack (kPa)	Tmax tack (°C)	T Range with Tack (°C)	Tack at 25 °C (kPa)	Work of Adhesion at 25 °C (J)
EBA	563	95	70–120	<10	<10
B2F20	219	75	20–55	151	38
B2F30	870	30	15–55	386	108
B2F43	1329	25	5–55	1329	456
B2F50	1294	45	20–55	153	38
B2F57	1553	45	20–55	147	37
B2F62	1636	40	25–55	180	36
